# A win-win scenario for photosynthesis and the plasma membrane H^+^ pump

**DOI:** 10.3389/fpls.2022.982485

**Published:** 2022-08-12

**Authors:** Satoru N. Kinoshita, Toshinori Kinoshita

**Affiliations:** ^1^Graduate School of Science, Nagoya University, Nagoya, Japan; ^2^Institute of Transformative Bio-Molecules (WPI-ITbM), Nagoya University, Nagoya, Japan

**Keywords:** leaves, pH homeostasis, photosynthesis, PM H^+^-ATPase, proton pump, transporters

## Abstract

In plants, cytosolic and extracellular pH homeostasis are crucial for various physiological processes, including the uptake of macronutrients and micronutrients, cell elongation, cell expansion, and enzyme activity. Proton (H^+^) gradients and the membrane potential are generated by a H^+^ pump consisting of an active primary transporter. Plasma membrane (PM) H^+^-ATPase, a PM-localized H^+^ pump, plays a pivotal role in maintaining pH homeostasis in plant cells and extracellular regions. PM H^+^-ATPase activity is regulated by protein abundance and by post-translational modifications. Several stimuli have been found to activate the PM H^+^-ATPase through phosphorylation of the penultimate threonine (Thr) of the carboxy terminus. Light- and photosynthesis-induced phosphorylation of PM H^+^-ATPase are conserved phenomena among various plant species. In this work, we review recent findings related to PM H^+^-ATPase regulation in the photosynthetic tissues of plants, focusing on its mechanisms and physiological roles. The physiological roles of photosynthesis-dependent PM H^+^-ATPase activation are discussed in the context of nitrate uptake and cytoplasmic streaming in leaves.

## Introduction

During photosynthesis plants capture energy from sunlight and convert carbon dioxide, into carbohydrate. Photosynthesis is a crucial physiological process for plant cells; the illumination of plant leaves induces numerous photosynthesis-dependent signaling pathways and maintains photosynthetic performance. Plant cell pH homeostasis is essential for various photosynthesis-induced physiological processes in plastids, as well as nutrient uptake *via* secondary transporters and channels across membranes. Plastidial pH homeostasis was recently reviewed by Trinh and Masuda (2022). Plant macronutrient availability greatly influences photosynthesis (Hester and Mendelssohn, 1990; Grossman and Takahashi, 2001). Therefore, pH homeostasis in plant cells promotes nutrient acquisition for both plant growth and the maintenance of photosynthesis.

**FIGURE 1:**
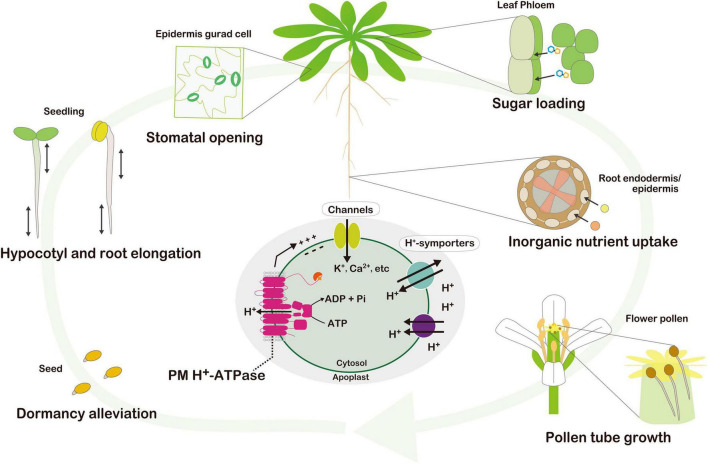
Physiological consequence of the PM H^+^-ATPase throughout the life of the plant. Seed dormancy alleviation, seedling hypocotyl and root elongation, stomatal guard cell opening, nutrient uptake in the root epidermis, sugar loading in phloem sieve elements, and flower pollen tube growth have been demonstrated to date. Active H^+^ pumping by PM H^+^-ATPase energizes the PM. Hyperpolarization and an H^+^ gradient across the PM, generated by H^+^ pumping, activate voltage-dependent channels and H^+^ symporters for substrate uptake.

Protons (H^+^) are translocated across the plasma membrane (PM) by PM H^+^-ATPase using energy provided by ATP hydrolysis. Many of the physiological roles of PM H^+^-ATPase in plants have been intensively studied such as stomatal opening in guard cells (Inoue and Kinoshita, 2017), seedling hypocotyl elongation (Hager, 2003), root elongation (Haruta et al., 2018), nutrient uptake by roots (Sondergaard et al., 2004), flower pollen tube growth (Hayashi and Palmgren, 2021), sugar loading in phloem sieve elements (DeWitt and Sussman, 1995), and seed dormancy alleviation (de Bont et al., 2019; Figure 1). The spatial and temporal regulation of PM H^+^-ATPase activity is critical for these physiological process. PM H^+^-ATPase activity is regulated by protein transcription and translation, as well as post-translational modification. Several environmental stimuli induce the phosphorylation of the penultimate threonine (Thr) on the carboxy terminus of PM H^+^-ATPase and subsequent binding of 14-3-3 protein to the region activate H^+^ pumping. The regulation of PM H^+^-ATPase activity by environmental stimuli and phytohormones was reviewed by Fuglsang and Palmgren (2021) and Miao et al. (2022); however, that of PM H^+^-ATPase activity in photosynthetic tissues is an emerging subject (Harada et al., 2002a; Okumura et al., 2012a,b). Therefore, we summarize recent findings regarding the regulatory mechanisms of PM H^+^-ATPase and its physiological roles in photosynthetic tissues.

## Photosynthesis-driven PM H^+^-ATPase activation *via* phosphorylation and its roles

### Photosynthesis-dependent activation of PM H^+^-ATPase in divergent plant species and tissues

Photosynthetically active radiation on photosynthetic tissues such as the thallus of *Marchatia polymorpha*, protonema of the moss *Physcomitrella*, and leaves of *Arabidopsis*, *Vallisneria*, rice, tobacco, and fava bean induces phosphorylation of the penultimate Thr of PM H^+^-ATPase (Okumura et al., 2012a,b, 2016; Harada et al., 2020). Two inhibitors of electron transport reactions in photosynthesis, 3-(3,4-dichlorophenyl)-1,1-dimethylurea (DCMU) and 2,5-dibromo-3-methyl-6-isopropyl-p-benzoquinone (DBMIB), have been shown to interrupt the light-induced phosphorylation of PM H^+^-ATPase (Okumura et al., 2012a,^2016^). Exogenous sugar supplementation to tissues in the dark induces the phosphorylation of PM H^+^-ATPase (Okumura et al., 2016). These results demonstrate that photosynthetic products are responsible for the activation of PM H^+^-ATPase *via* phosphorylation. Sucrose supplementation to *Arabidopsis* seedlings induces phosphorylation of the penultimate Thr of PM H^+^-ATPase, suggesting that the photosynthetic product-dependent phosphorylation of PM H^+^-ATPase is not limited to leaves (Niittylä et al., 2007).

### Involvement of glycolysis and downstream metabolism in PM H^+^-ATPase phosphorylation regulation

Kinoshita et al. (2022) recently reported that glycolysis inhibition by a glucose analog, 2-deoxy glucose, suppresses the photosynthetic product-induced phosphorylation of PM H^+^-ATPase in *Arabidopsis* leaves, suggesting the involvement of glycolysis and downstream metabolism. As cytosolic ATP and NAD(P)H are end-products of glycolysis, respiration, and photosynthesis, it raised a question: whether the glycolysis and downstream metabolites are the direct stimuli on PM H^+^-ATPase, or the end-products are the key stimuli. Recent advance in biosensors of ATP/NADPH and a study using inducible silencing of specific metabolic enzyme may reveal an interesting connection between photosynthesis and glycolysis or respiration related to PM H^+^-ATPase regulation in leaves.

In addition, it is interesting to note that carbon availability and intact glycolysis are responsible for PM H^+^-ATPase activation in yeast (Mazón et al., 2015). Although the regulatory carboxy-terminus of PM H^+^-ATPase in yeast is shorter and structurally different compared to plant PM H^+^-ATPase, the response to carbon availability in cell is conserved phenomena. Taken together with the case of plant PM H^+^-ATPase activation in leaves, it would be more interesting to investigate what molecular components regulate PM H^+^-ATPase in response to carbon availability in cell, using both yeast and plants as the model.

## Physiological significance of PM H^+^-ATPase in illuminated leaves

### PM H^+^-ATPase activation may increase nitrate uptake in leaves

Maintaining the H^+^ gradient and membrane potential across the PM is essential for macronutrient and micronutrient uptake because numerous PM-localizing H^+^ symporter and voltage-dependent channels are involved in nutrient uptake from extracellular regions. The involvement of PM H^+^-ATPase in nutrient uptake was reviewed by Sondergaard et al. (2004). A mathematical model of the membrane transporter system, including PM H^+^-ATPase, was developed for the simulation of active coupling of H^+^ symporters and the H^+^ pump (Dreyer, 2021).

The putative physiological roles of photosynthesis-dependent PM H^+^-ATPase activation may be summarized by focusing on the relationship between PM H^+^-ATPase and macronutrient nitrate in leaves. The molecular mechanism of nitrate uptake in roots has been well studied because this process is the greatest limitation on plant nitrate acquisition (Xu et al., 2012). In *Arabidopsis*, concentrated nitrate is translocated from roots to the leaf apoplast *via* the xylem stream (Dechorgnat et al., 2011). However, nitrate import and translocation to leaves are important limitations on plant shoot growth (Chiu et al., 2004; Hsu and Tsay, 2013). Photosynthetic tissues generally invest large amounts of nitrogen in the synthesis of photosynthesis-related proteins, including rubisco, a light-harvesting complex (Evans and Clarke, 2019). Nitrate reduction in illuminated leaves is the key driving force for nitrate uptake to mesophyll cells (Cookson et al., 2005). Thus, mesophyll cells may require active nitrate uptake from the apoplast to compensate nitrogen availability during photosynthesis. Light-dependent nitrate uptake in roots and leaves has been suggested in soybean (Delhon et al., 1995). The involvement of the PM membrane potential in light-dependent nitrate uptake has been demonstrated using microelectrode recordings in *Arabidopsis* leaves (Cookson et al., 2005). Disks from nitrogen-starved cucumber leaves showed pH- and light-dependent increases in nitrate content, suggesting the involvement of PM H^+^-ATPase (Nikolic et al., 2012). Light- and PM H^+^-ATPase activation-induced nitrate uptake was also recently reported in *Arabidopsis* leaves grown on soil without nitrate starvation (Kinoshita et al., 2022).

**FIGURE 2:**
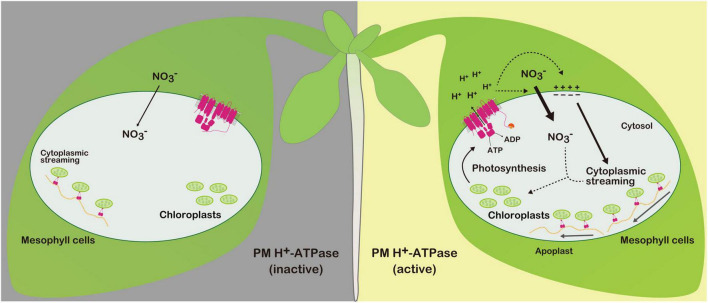
Photosynthesis-induced PM H^+^-ATPase activation and its physiological roles in mesophyll cells. Photosynthesis activates PM H^+^-ATPase *via* phosphorylation of the penultimate threonine (Thr). Activated PM H^+^-ATPase increases nitrate influx and cytoplasmic streaming under light illumination. Efficient nitrate influx and redistribution of chloroplast *via* cytoplasmic streaming by organelle-associated actin/myosin system may support photosynthesis. Under overnight dark conditions, the penultimate Thr of PM H^+^-ATPase is dephosphorylated, thereby inactivating PM H^+^-ATPase.

Leaf nitrogen availability greatly influences photosynthetic activity (Makino, 2011). Therefore, we speculate that photosynthesis activates PM H^+^-ATPase to compensate the nitrate pool, which is substantially reduced and acclimated during light illumination (Figure 2). Future studies should track nitrate flux within the cell to improve our understanding of the positive relationship between photosynthesis and PM H^+^-ATPase.

### PM H^+^-ATPase activation may increase cytoplasmic streaming

Because photosynthesis-dependent PM H^+^-ATPase activation is a conserved phenomenon across numerous plant species, it is valuable to dissect the physiological roles of PM H^+^-ATPase in photosynthetic tissues using different types of plant species. For example, mesophyll cells of the aquatic monocot *Vallisneria* show cytoplasmic streaming and active H^+^ pumping under red light illumination (Harada et al., 2002a,b). Subsequently, phosphorylation of the penultimate Thr of PM H^+^-ATPase was confirmed to be responsible for light-induced H^+^ pumping, and cytoplasmic streaming was revealed to be dependent on the H^+^ pumping (Harada et al., 2020). Cytoplasmic streaming is thought to be generated by association of myosin/actin system with organelle (Shimmen and Yokota, 2004). Modified cytoplasmic streaming *via* introducing improved myosin XI into Arabidopsis has been suggested to contribute to regulating cell size (Tominaga and Ito, 2015), which implies that constant redistribution of chloroplasts *via* light-induced cytoplasmic streaming contributes to cell growth by facilitating photosynthesis (Figure 2). Considering that PM H^+^-ATPase activity is also important for cell elongation and expansion in various plant species, it would be interesting to investigate the evolution of the mechanism by which photosynthesis controls divergent physiologies through the regulation of PM H^+^-ATPase activity.

## Conclusions and perspectives

In this review, the relationship between photosynthesis and PM H^+^-ATPase was discussed in the context of the positive roles of active PM H^+^-ATPase in mesophyll cells. Photosynthesis energizes the PM, and therefore may activate nitrate uptake and cytoplasmic streaming to maintain efficient photosynthesis (Figure 2).

Macronutrient and micronutrient availability (i.e., nitrogen, phosphorus, potassium, and magnesium) greatly affects photosynthesis activity (Makino, 2011; Tränkner et al., 2018; Sitko et al., 2019). The uptake of these molecules may be facilitated by the H^+^ gradient or membrane potential. Thus, the physiological roles of photosynthesis-dependent PM H^+^-ATPase in macronutrient and micronutrient uptake should be further investigated in the context of a win-win scenario for photosynthesis and the H^+^ pump. Notably, the H^+^ pump, photosynthesis, nutrient acquisition, and cytoplasmic streaming are conserved in numerous plant species. Therefore, further investigation of the molecular mechanisms underlying these conserved phenomena will open new avenues for the elucidation of these evolutionary questions.

## Author contributions

SNK and TK designed the concept. SNK wrote the manuscript and drew the figures. Both authors reviewed and approved the final version of the manuscript.
